# Amygdala-orbitofrontal structural and functional connectivity in females with anxiety disorders, with and without a history of conduct disorder

**DOI:** 10.1038/s41598-018-19569-7

**Published:** 2018-01-18

**Authors:** Philip Lindner, Pär Flodin, Peter Larm, Meenal Budhiraja, Ivanka Savic-Berglund, Jussi Jokinen, Jari Tiihonen, Sheilagh Hodgins

**Affiliations:** 1 Department of Clinical Neuroscience, Karolinska Institutet, Stockholm, Sweden; 2 Stockholm Center for Dependence Disorders, Stockholm County Council, Stockholm, Sweden; 3 Department of Psychology, Stockholm University, Stockholm, Sweden; 4 Umeå Center for Functional Brain Imaging, Umeå University, Umeå, Sweden; 5 Center for Aging and Demographic Research, Umeå University, Umeå, Sweden; 6 Centre for Clinical Research, Uppsala University, Uppsala, Sweden; 7 Department of Women’s and Children’s Health, Karolinska Institutet, Stockholm, Sweden; 8 Neurology Clinic, Karolinska University Hospital, Huddinge, Sweden; 9 Department of Clinical Sciences, Umeå University, Umeå, Sweden; 10 Department of Forensic Psychiatry, University of Eastern Finland, Niuvanniemi Hospital, Kuopio, Finland; 11 Département de Psychiatrie, Université de Montréal, Montréal, QC Canada

## Abstract

Conduct disorder (CD) and anxiety disorders (ADs) are often comorbid and both are characterized by hyper-sensitivity to threat, and reduced structural and functional connectivity between the amygdala and orbitofrontal cortex (OFC). Previous studies of CD have not taken account of ADs nor directly compared connectivity in the two disorders. We examined three groups of young women: 23 presenting CD and lifetime AD; 30 presenting lifetime AD and not CD; and 17 with neither disorder (ND). Participants completed clinical assessments and diffusion-weighted and resting-state functional MRI scans. The uncinate fasciculus was reconstructed using tractography and manual dissection, and structural measures extracted. Correlations of resting-state activity between amygdala and OFC seeds were computed. The CD + AD and AD groups showed similarly reduced structural integrity of the left uncinate compared to ND, even after adjusting for IQ, psychiatric comorbidity, and childhood maltreatment. Uncinate integrity was associated with harm avoidance traits among AD-only women, and with the interaction of poor anger control and anxiety symptoms among CD + AD women. Groups did not differ in functional connectivity. Reduced uncinate integrity observed in CD + AD and AD-only women may reflect deficient emotion regulation in response to threat, common to both disorders, while other neural mechanisms determine the behavioral response.

## Introduction

Conduct disorder (CD) indexes a childhood or adolescent onset of antisocial behavior, ranging from lying, truancy and rule-breaking, to serious offences such as physical and sexual assault^1^. CD is associated with a wide range of adverse outcomes in adulthood, including educational failure, unemployment, violent and non-violent criminality, substance dependence and other mental health problems^2–4^. In females, CD prevalence estimates range from 0–1.4%, and the clinical phenotype is distinctive, particularly with respect to age of onset and frequency and types of aggressive behavior^5^. Girls with CD, or prior CD, give birth at a young age^6^, provide non-optimal parenting^7^, including physical maltreatment^3^, to their offspring who are at increased risk of conduct problems^8,9^. Despite the elevated rates of multiple negative outcomes in adulthood for women who had presented CD in childhood/adolescence, and for their offspring, little is known about such women and the neurobehavioral mechanisms that underlie their antisocial behaviors, nor about effective treatment.

Among children and adolescents with CD and adults with prior CD and subsequent Antisocial Personality Disorder (ASPD), comorbid anxiety disorders (ADs) are common. Among children/adolescents with CD, ADs are presented in 22–33% in community samples and 60–75% of those who seek treatment^10–12^. This comorbidity between CD and AD may emerge as early as 24 months^13^, and there is evidence to suggest both that ADs precede conduct problems and that CD precedes ADs^14^. Studies of large community samples of adults and of prisoners show that approximately half of those with ASPD also present an AD^15–17^. Among children and adolescents with CD^10,11^, and among adult offenders with ASPD^17^, those with and without comorbid ADs present similar levels of aggressive behavior and violent criminality.

The Research Domain Criteria (RDOC) for the classification of mental disorders was designed, in part, because psychiatric syndromes appearing clinically distinct may result from the same etiology^18^. There is a striking similarity in the neuro-behavioral mechanisms thought to underlie CD/ASPD and AD, including hostile attribution bias and autonomic hyper-arousal^14^. Further elucidating these mechanisms may inform cognitive behavioral therapies (CBT) targeting common or unique factors in adolescents and adults with CD/ASPD and ADs. The majority of individuals with CD/ASPD present no, or low, psychopathy traits^19^. Hyper-sensitivity to threat (i.e. hostile attribution bias)^20^ drives their antisocial behavior and reactive aggression. These behaviors are further promoted by impulsivity and impaired emotion regulation^21^, including poor anger control^22^ that mediates reactive aggression^23^. Individuals with ADs show similar attention biases^24,25^. Additionally, robust evidence from meta-analyses confirms that individuals with AD-only^26,27^, and those presenting early-onset stable antisocial behavior (without psychopathy) display hyper-activation of the amygdala when viewing emotional faces^28–31^.

This evidence suggests that both CD and ADs are associated with dysfunctional regulation of amygdala reactivity to threat by the orbitofrontal cortex (OFC)^32^. Deficient down-regulation of the amygdala by the OFC could be due, at least in part, to abnormalities of the white matter tract, the uncinate fasciculus (UF), that connects these two regions^33^. In healthy adults, greater structural integrity of the UF has been associated with greater attention bias to nonconscious threat^34^ and greater self-reported use of reappraisal to regulate emotions^35,36^. Among male adolescents with CD, several studies have observed UF abnormalities^37–39^. However, these studies have not investigated comorbid ADs, despite evidence of UF abnormalities among individuals with AD-only, including social anxiety disorder^40–42^ and generalized anxiety disorder^43^. Additionally, in healthy men and women, stronger UF integrity has been associated with both lower^36,44,45^ and higher trait anxiety levels^46,47^. Similarly, reduced resting-state amygdala-frontal functional connectivity has been observed both among individuals presenting antisocial behavior^48^ and individuals with ADs only^49,50^.

The reasons for the elevated prevalence of ADs among children, adolescents, and adults engaging in antisocial behavior remain unknown. Both antisocial disorders and ADs are characterized by hyper-sensitivity to threat and hostile attribution bias. The two disorders differ, dramatically, however in their response to threat, with antisocial individuals engaging in reactive aggressive behavior and individuals with ADs presenting a pattern of avoidance. The evidence that both disorders are characterized by similar structural abnormalities is not clear because studies of antisocial samples have either excluded participants with comorbid ADs^37,38^, making the samples unrepresentative, or matched groups on anxiety^39^. Further, most of these studies have focused on males or mixed-sex samples. ADs are twice as common among females than males^51^, while CD/ASPD, and in particular aggressive behavior, is less common among females than males^5^. In addition, there are important sex differences in emotional processing^52^, associated limbic volumes^53^, and whole-brain structural and functional connectivity^54,55^. To the best of our knowledge, no study has compared the neural correlates of ADs and the more typical form of antisocial behavior with no or low psychopathic traits, indexed by diagnoses of CD or ASPD.

### The present study

The present study focused on a clinical sample of participants in an effort to determine whether a common mechanism promoted symptoms of both CD + AD and AD, and to explore factors associated with behavioral differences in the two disorders. Consistent with the RDOC framework^18^, we studied presumed shared and distinct neural mechanisms in an effort to provide findings that may inform treatments, and also explain the high comorbidity of CD and AD. We hypothesized that young adult women with a history of CD and comorbid AD (CD + AD) would show the same structural abnormalities of the UF as those with AD-only, and similarly reduced amygdala-OFC resting-state functional connectivity, compared to women with neither disorder (ND) but well-matched on other clinical characteristics. Given the high levels of depression associated with ADs^56^, of substance use disorders associated with CD^57,58^, and of childhood maltreatment associated with both disorders^59^, group comparisons were adjusted for these comorbid disorders and trauma experiences in an effort to disentangle observed associations between diagnoses and brain measures.

While individuals with CD and those with AD display hyper-reactivity to threat, their behavioral responses to threat differ. Individuals presenting CD + AD engage in both approach (aggression) and avoidance behaviors, while those with AD-only respond with avoidance behaviors. Consequently, we conducted exploratory analyses to determine whether any impaired structural integrity of the UF would be associated with different factors among participants with CD + AD and AD-only. The temperament trait of Harm Avoidance (HA) indexes susceptibility to fear and anxiety and the tendency to react with inhibitory, avoidant, behaviors^60^. HA has consistently been found to be elevated in adolescents and adults with ADs^61^, and either unrelated^62^ or negatively associated with antisocial behavior^63^ (unadjusted for psychopathic traits^64^). Among healthy adults, HA has been associated with increased amygdala reactivity^65–67^, amygdala resting-state connectivity^68^, and amygdala volume^69^, as well as both increased and decreased UF structural integrity^70–72^. We thus hypothesized that HA would be associated with UF integrity among participants with AD-only and not among those with CD + AD. We reasoned that by contrast, among participants with CD + AD the UF abnormality would be associated with the combination of current anxiety symptoms (importantly, not avoidant behavior, as indexed by HA scores) and poor anger control, since this group presents both anxiety and aggressive behavior.

## Material and Methods

### Sample

Women were recruited from a longitudinal study of adolescents who consulted for substance misuse at a specialized clinic in Stockholm^73–76^. Thirty-nine ex-clients had been assessed at baseline, and 6, 12, 60 and 78 months later. In the larger cohort of female ex-clients, 58% met criteria for a substance use disorder (SUDs) at baseline^77^. Only 53.8% had received treatment-as-usual for SUDs by the 60-month follow-up, and this treatment was found to be unsuccessful in preventing persistence of SUDs in a five-year follow-up study^73^. At the 60-month follow-up, 31 sisters of clients treated at the clinic were recruited. These 70 women underwent Magnetic Resonance Imaging (MRI) at the 78-month follow-up. Participants were divided into three groups: (a) at any assessment met criteria for AD and presented CD (CD + AD, n = 23); (b) at any assessment met criteria for AD but not for CD (AD-only, n = 30) or (c) never met criteria for either AD or CD (neither disorder, ND, n = 17). Comparing the CD + AD and AD-only groups to a clinical comparison group (ND) was preferred over comparison to healthy women since a previous study on an overlapping sample^74^ indicated brain-wide, regionally unspecific decreased axial diffusivity in CD women as compared to healthy women. Finding UF abnormalities in the CD + AD and AD-only groups compared the ND group, thus strengthens the claim of the regional specificity of these abnormalities to the UF. We were unable to form a CD-only group, as 11 of the 17 females who presented CD and no AD at baseline^77^ had developed an AD by the 78 month follow-up.

No participant reported any neurological disorders, loss of consciousness for more than 30 minutes, or any other contra-indication for a MRI brain scan. Participants were asked to refrain from alcohol and drug use for three days prior to scanning. Using a breath analyzer and saliva sample, participants were screened for recent use of alcohol and seven classes of illegal drugs. None tested positive. Four participants reported taking psychoactive medication (anxiolytic, antidepressant, stimulant, hypnotic or antipsychotic) in the days prior to the scan. See Table 1 for sample characteristics.

**Table 1:** Sample characteristics.

Measure	Neither disorder (ND; n = 17)	AD-only group (n = 30)	CD + AD group (n = 23)	Statistics
Mean age (SD)	24.78 (4.33)	25.23 (2.92)	23.71 (2.92)	F[2,67] = 0.954, p = 0.390
Mean performance IQ (SD)	11.24 (2.81)	10.73 (2.85)	9.13 (2.98)	F[2,66] = 3.01, p = 0.056
Mean verbal IQ (SD)	10.00 (1.90)	9.23 (2.34)	7.86 (2.66)	F[2,66] = 4.24, p = 0.019 Post-hoc: ND > CD + AD
Mean BAI score (SD)	4.88 (4.30)	5.93 (4.76)	11.04 (10.66)	F[2,67] = 4.64, p = 0.013 Post-hoc: CD + AD > (ND&AD)
% Any recent aggressive behavior	23.5%	23.3%	34.8%	FET, p = 0.585
% Lifetime major depression	11.8%	70.0%	56.5%	FET, p < 0.001
% Lifetime alcohol dependence	23.5%	23.3%	39.1%	FET, p = 0.443
% Lifetime drug dependence	4.9%	23.3%	43.5%	FET, p = 0.027
% Maltreatment before age 16^†^	0%	19%	50%	FET, p = 0.0469

FET: Fisher’s exact test. ^†^Maltreatment data omitted case-wise for n = 3 in AD-only and n = 5 in CD + AD group due to being inconclusive as to what age maltreatment occurred.

### Procedure

Details of procedures of previous assessment waves have been reported^73,77,78^. The current study, and all previous assessment waves were approved by the Regional Ethics Review Board in Stockholm and complied with the Helsinki declaration. Participants were invited by telephone and mail to participate in the 78-month follow-up. Clinical assessment and MRI was completed in a single session at the Karolinska University Hospital. The datasets generated during and/or analyzed during the current study are available from the corresponding author on reasonable request. Participants provided written informed consent and were provided with 1600 SEK in gift certificates for their participation.

### Measures

#### Clinical assessment

Clinical assessments at each wave of data collection were conducted by clinicians trained to use each of the validated, structured, diagnostic tools. At all assessments, ADs, CD and other mental disorders were assessed using the Schedule for Affective Disorders and Schizophrenia for School-Age Children^79^ (if under age 18) or the Structured Clinical Interview for DSM IV (SCID)^80^ (if 18 or older). ADs were defined to include specific phobia, substance-induced anxiety disorder, social anxiety disorder, post-traumatic stress disorder, panic disorder, obsessive compulsive disorder, generalized anxiety disorder, agoraphobia, anxiety disorder due to somatic illness, and anxiety disorder not otherwise specified. The prevalence of each AD within each group is presented in Supplementary Table S1. On the day of the scan, in addition to completing a diagnostic interview following the SCID, participants completed the Beck Anxiety Inventory (BAI)^81^ to assess current anxiety symptoms.

#### Intelligence

Verbal (VIQ) and performance (PIQ) intelligence quotients were estimated using the Vocabulary and Block design tests of the Wechsler Adult Intelligence Scale III^82^ at the 60-month follow-up.

#### Maltreatment

At study entry and at the 60 month follow-up, participants completed the Conflict Tactics Scale^83^ to report on physical abuse before age 16. Responses were dichotomized as none/minor, or severe/extreme (see supplementary material of Lindner *et al*. (2016) for details).

#### Harm Avoidance temperament traits

The Junior Temperament and Character Inventory (JTCI)^84^ was completed by the 39 ex-clients at baseline when they were, on average, 16 years old. Scores for the Harm Avoidance (HA) subscale^60^ were calculated according to the canonical procedure. The psychometric validity of the JTCI has been previously demonstrated in the larger cohort that included these 39 participants^62^.

#### Poor anger control

Poor anger control in adulthood was assessed in all participants using the Psychopathy Checklist: Revised (PCL-R)^85^ and the corresponding item that was completed 18 months prior to scan. All items on the PCL:R were scored by a trained interviewer based on a semi-structured interview schedule, as well as all other available information sources. Item scores were dichotomized as either zero or higher (poor anger control).

### MRI acquisition

Scanning was performed using a 3-Tesla MRI scanner (MR750 GE Healthcare, Milwaukee, WI, USA) with an eight-channel array coil (*in-Vivo*, Gainesville, FL, USA). Diffusion-weighted data were acquired using a single-shot, echo planar imaging, twice-refocused spin-echo diffusion pulse sequence across 60 noncollinear directions with b = 1000, along with eight initial b = 0 directions. Field-of-view was 23 cm, acquisition matrix 116 × 116 and slice thickness 2 mm, providing 2-mm isotropic resolution. Echo time was 81.6 ms and repetition time 7600 ms. Preprocessing of raw, diffusion-weighted images was performed using DTIPrep^86^ which automatically removes low-quality directions, corrects for eddy-currents and motion, and adjusts the gradient table values.

During acquisition of resting-state data, participants were instructed to stay awake and focus on a white crosshair on a black background on a screen mirrored above their head. This procedure was preferred over closed-eye acquisition to minimize confounding effects of fatigue and sleep. Image parameters were: flip angle, 90°; repetition time, 2.5 s, echo time, 30 ms; field of view, 288 mm^2^; slice thickness, 3 mm. Also acquired were high-resolution (1 mm^3^), fast-spoiled T1-weighted anatomical images in the axial plane with a 12° flip angle; echo time, 3.1 ms; repetition time, 7.9 ms; and 176 slices, for structural registration of connectivity data.

### Tractography

**Figure 1:**
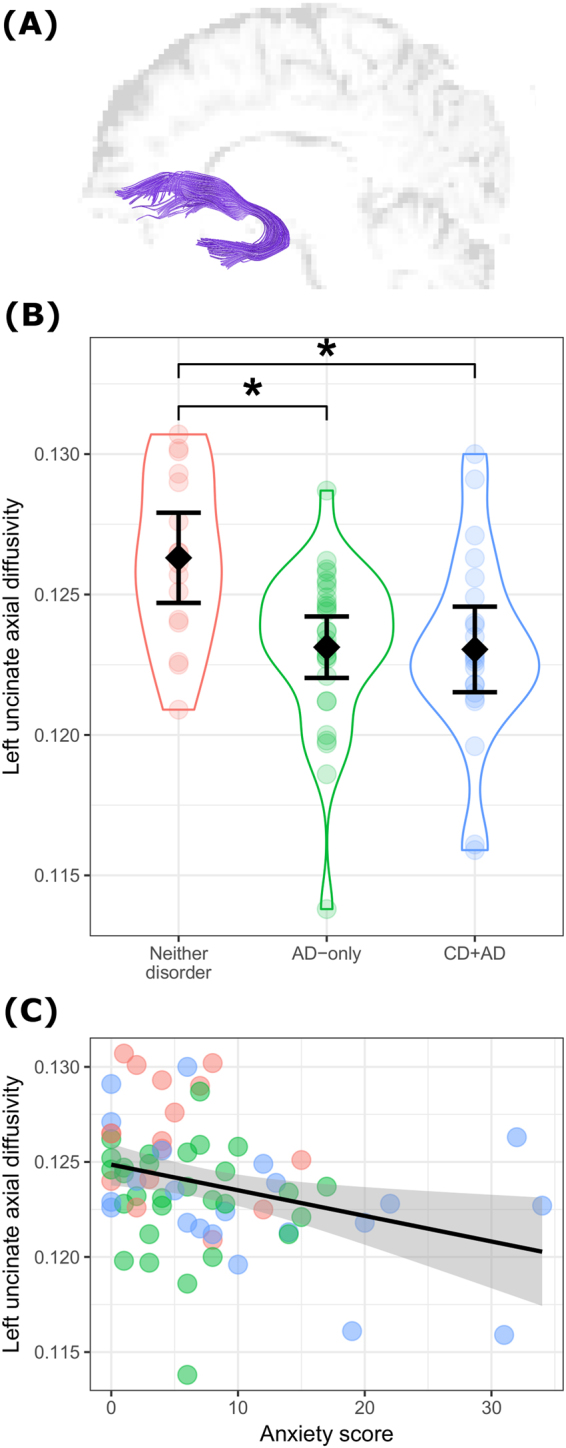
Uncinate fasciculus findings. (**A**) Example dissection of the left uncinate. (**B**) Between-group differences in left uncinate AD (group means and 95% confidence intervals superimposed on grouped individual scatter). (**C**) Significant correlation between current anxiety score and left uncinate AD in the whole sample.

Due to the shape and location of the UF (which intersects both with the inferior fronto-occipital tract in the OFC and the inferior longitudinal tract in the temporal pole), voxelwise methods that rely on inter-subject normalization, such as Tract-Based Spatial Statistics^87^ used in our previous study, contrasting whole-brain white matter integrity between CD and healthy women^74^, are limited in their abilities to properly delineate the UF. Instead, whole-brain tractography and manual, virtual dissection was employed^76^. Preprocessed diffusion-weighted data were tensor-fitted in Diffusion Toolkit and whole-brain tractography performed using the interpolated streamline algorithm, an angle threshold of 34° and a standard FA interval of 0.2–1. Dissections of the left and right UF were performed using Trackvis and a validated dissection protocol^88^ that included placing a region-of-interest (ROI) in the OFC extending to the external capsule, visualizing all tracts passing through this ROI, then placing a second, AND-gated ROI in the temporal pole. This method captured both branches of the UF: the antero-medial branch terminating in the medial frontal pole, and the ventro-lateral branch that terminates in lateral OFC^89^. Spurious reconstruction-artefact tracts were manually removed using NOT-gated ROIs. See panel A of Fig. 1 for an example dissection. Delineated left and right UF tract maps were saved for each participant and used to extract average tract axial diffusivity, fractional anisotropy (FA) and radial diffusivity (RD) values. In three cases, the left UF could not be reliably delineated; these were omitted case-wise in analyses.

### Resting-state connectivity analyses

Resting-state connectivity data were preprocessed and analyzed using the CONN toolbox^90^ running on SPM12. Preprocessing steps included: slice timing correction, realignment to the mean image (motion correction), anatomical-functional co-registration, tissue segmentation, direct normalization into MNI space, and 8 mm Gaussian smoothing. Data was then band-pass filtered (0.008–0.09 Hz, after nuisance regression). Nuisance regressors included 6 realignment parameters, 5 principal components from white matter signals and CSF, respectively, using a principal component (PCA) based noise correction approach. Additionally, volumes exceeding 0.5 mm frame wise displacement or 3 standard deviation global signal intensity change were regressed out. Seed regions were defined according to the 3 mm, 90 ROI Automated Anatomical Labeling Atlas^91^. To cover both the antero-medial and ventro-lateral branches of the OFC, separate bilateral seeds covering the orbitofrontal parts of the inferior and middle frontal gyri (respectively) were included, as well as the amygdala. Time series from these ROIs were extracted and used to calculate inter-ROI correlations.

### Statistical analyses

A power analysis indicated that a large omnibus difference between the three groups (f > 0.45) could be detected in an ANOVA model with 80% power and 17 participants per group, and that a t-test between the AD-only and CD + AD groups could detect a large pairwise difference of Cohen’s d > 0.8.

Initially, extracted structural and functional connectivity metrics were compared across the three groups (AD-only, CD + AD, ND) using ANOVA with Bonferroni-corrected post-hoc tests and bootstrapped 95% confidence intervals of the mean differences. Next, these analyses were adjusted for VIQ, PIQ, lifetime major depression, alcohol dependence, drug dependence, and childhood maltreatment in an ANCOVA model. In a final step, significant metrics were correlated with current anxiety symptoms.

## Results

### Structural connectivity

As shown in panel B of Fig. 1, there was a significant group difference in left UF axial diffusivity (F[2,64] = 6.55, p = 0.0026). Pairwise t-tests revealed that both the AD (p_Bonf_ = 0.0048; ∆M = 0.003 [95% CI: 0.001–0.005]) and CD + AD (p_Bonf_ = 0.0064; ∆M = 0.003 [95% CI: 0.001–0.005]) groups displayed lower UF axial diffusivity compared to the ND group, with no difference between AD and CD + AD groups (p_Bonf_ = 1; ∆M = 0.0001, [95% CI: −0.002–0.002]). After adjusting for major depression, alcohol dependence, drug dependence, VIQ, and maltreatment, the main effect of group remained significant (F[2,51] = 5.56, p = 0.00065) with other factors non-significant (all p > 0.19). There were no group differences in any other UF metric. As illustrated in panel C of Fig. 1, current anxiety symptoms were negatively correlated with left UF axial diffusivity (r = −0.31, n = 67, p = 0.011), and at a trend level with right UF axial diffusivity (r = −0.21, n = 67, p = 0.087). Anxiety symptoms were not correlated with FA or RD.

### Functional connectivity

**Figure 2:**
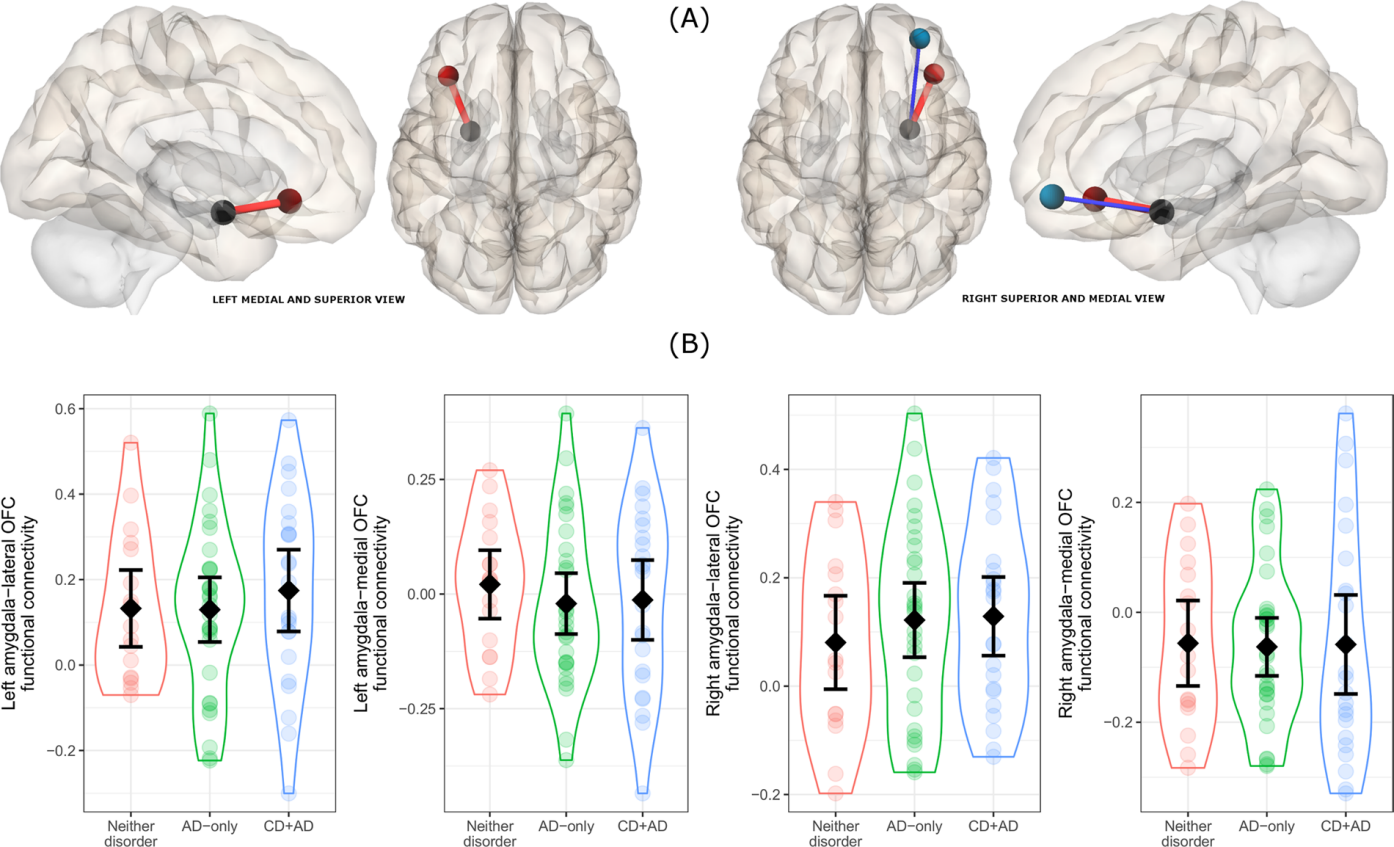
Functional connectivity results. (**A**) Positive (red) and negative (blue) associations between left and right amygdala and antero-medial and lateral OFC targets seeds (non-significant left amygdala-lateral OFC association not shown). (**B**) No significant between-group differences in any functional connectivity measure (group means and 95% confidence intervals superimposed on grouped individual scatter).

As illustrated in panel A of Fig. 2, in the left hemisphere, amygdala activity was positively associated with lateral OFC activity (t = 6.03, p < 0.001), but there was no association with activity of the antero-medial OFC (t = −0.38, p = 0.70-). In the right hemisphere, amygdala activity was positively associated with lateral OFC activity (t = 5.51, p < 0.001) and negatively associated with antero-medial OFC activity (t = −3.02, p = 0.004). As illustrated in panel B of Fig. 2, there was no overall difference in any functional connectivity metric between the three groups. Excluding participants taking psychoactive medication did not alter results. However, examining the whole sample revealed that participants with a current anxiety disorder (n = 6 AD-only and n = 9 CD + AD) showed reduced connectivity between the left amygdala and left lateral OFC (t = −2.44, p = 0.017). This difference remained when excluding the participants taking psychoactive medication (t = −2.40, p = 0.019). There was no association between current anxiety symptoms and left amygdala to lateral OFC connectivity, either in the whole sample (t = −0.57, p = 0.57) or among those with a current anxiety disorder (t = 1.64, p = 0.12).

### Functional and structural connectivity

There were no significant within-hemisphere correlations between any structural and functional connectivity measures.

### Exploratory analyses

As hypothesized, participants with CD + AD and AD-only, as compared to those with ND, displayed similar reductions of UF structural integrity. Both CD and AD are characterized by hyper-reactivity to threat. However, behavioral responses to threat differ in the two disorders. Individuals presenting CD + AD engage in both approach (aggression) and avoidance behaviors, while those with AD-only respond with avoidance behaviors. Consequently, we reasoned that the impaired structural integrity of the UF would be associated with different factors among participants with AD-only and those with CD + AD.

**Figure 3:**
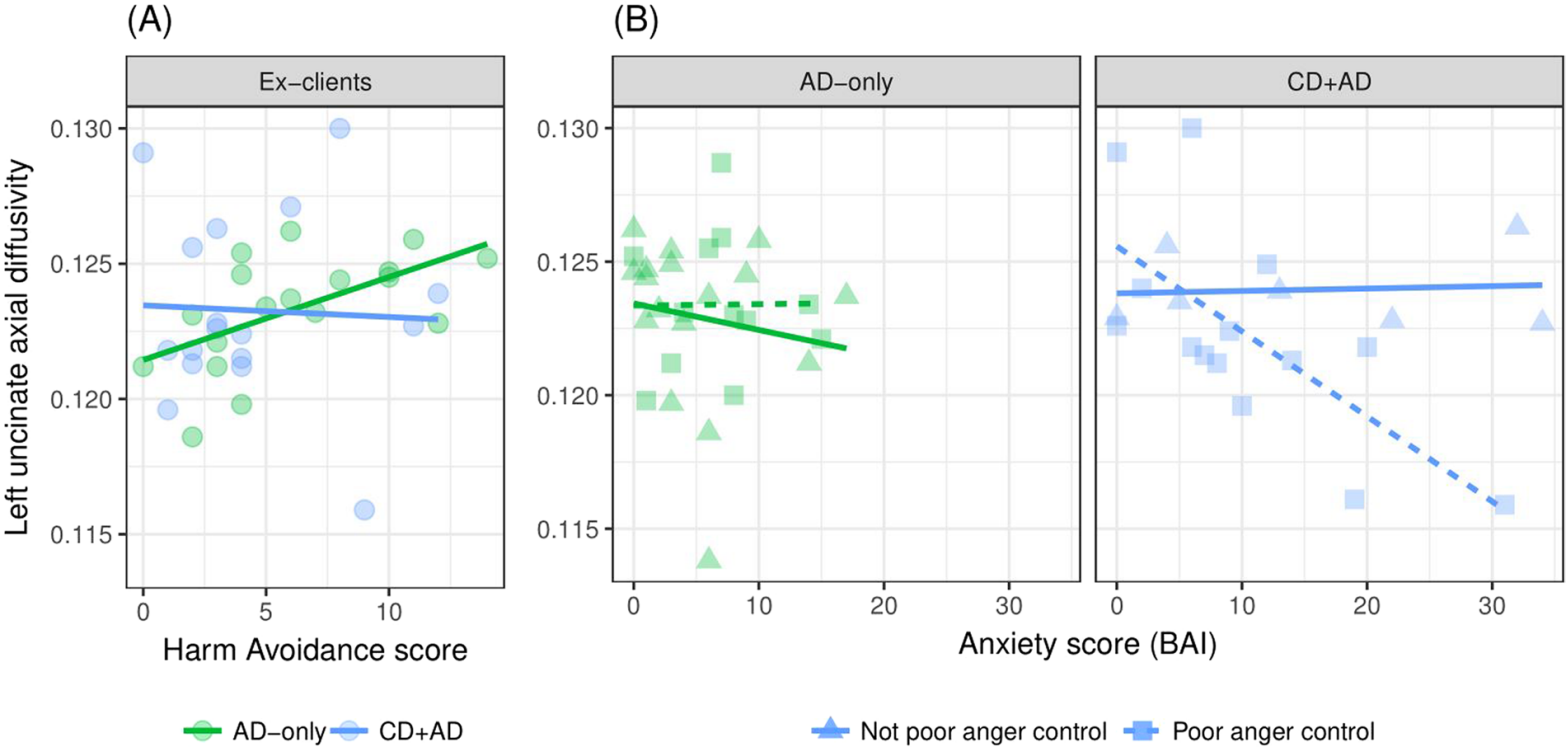
Disorder-distinct association of uncinate integrity with Harm Avoidance traits and the interaction of anger control ability and anxiety. (**A**) A significant correlation between Harm Avoidance traits (measured in adolescence) in ex-clients with AD-only, but not CD + AD. (**B**) A significant interaction effect of poor anger control and anxiety scores in participants with CD + AD (right panel) but not AD-only (left panel).

Analyses were computed within each group (AD-only and CD + AD) separately. As illustrated in panel A of Fig. 3, there was a positive association between HA scores and left UF axial diffusivity (r = 0.57, p = 0.013) among AD-only participants (r = 0.57, p = 0.013), and not among those with CD + AD (r = −0.04, p = 0.866). Next, multiple regression models were computed to determine whether UF axial diffusivity was associated with anger control, current anxiety symptoms, or the interaction of anxiety symptoms and anger control within each group. As seen in panel B of Fig. 3, among those with CD + AD, only the interaction term was significant (t = −2.797, p = 0.012) such that anxiety symptoms were negatively associated with axial diffusivity of the UF only among participants with poor anger control. By contrast, among participants with AD-only, neither anxiety symptoms, anger control, nor the interaction term was associated with UF axial diffusivity.

## Discussion

This is the first study to directly compare structural and functional amygdala-frontal connectivity of women with AD-only and CD + AD and those with neither disorder. Among young adult women, those with a history of ADs and those with a history of both CD and ADs showed similarly reduced structural integrity of the UF compared to women with neither disorder. Importantly, this abnormality was not associated with comorbid depression, alcohol dependence, drug dependence, childhood maltreatment, or IQ. The most parsimonious interpretation of these results is that the abnormality of the UF is a marker of lifetime AD, consistent with reports of reduced structural integrity of the UF in AD-only samples^40–42^, and with correlations between UF structural integrity and trait anxiety in non-clinical samples^36,44–47^. As previously noted, studies that reported a similar abnormality in antisocial individuals did not attempt to disentangle the impact of ADs or anxiety symptoms. We were unable to include a CD-only group in the present study, as all but six of the females with CD that we recruited as adolescents when they sought treatment for substance misuse initially presented a comorbid AD or developed an AD during the subsequent seven years.

While reduced structural integrity of the UF characterized both women with AD and those with CD + AD, the correlates of UF integrity differed in the two groups. In the AD-only group, UF integrity was positively associated with Harm Avoidance temperament scores, while in the CD + AD group, the combination of current anxiety symptoms and poor anger control was negatively associated with UF integrity. It could be that the abnormality of the UF explains, at least in part, the hypervigilance to threat and poor emotional regulation characterizing both CD and AD, while other factors and associated neural alterations are associated with the distinct response to threat shown by each disorder. If this speculative interpretation is correct, it would mean that a common neural mechanism underlies both AD and CD, manifested as reduced integrity of the UF, and that other mechanisms, unique to each disorder, underlie the pattern of behavioral responses to threat.

Evidence indicates that amygdala-OFC circuitry plays a key role in emotion regulation, reward processing, and reversal learning^92^. Temporal discounting, that is choosing a delayed, larger, reward rather than a smaller immediate one, has been positively associated with FA of the UF in healthy individuals^93^. A failure to delay rewards has been observed among individuals with ADs and among those with CD^94,95^. These findings are consistent with the preference shown by anxious individuals to avoid exposing themselves to the stressor (immediate negative reinforcement) over the long-term reward of extinction through exposure. Failure to delay gratification is also associated with antisocial behavior and criminality^96^.

Both ADs and antisocial behavior are characterized by hyper-reactivity to perceived threat^20,24,25^, and poor emotion regulation. The self-reported use of the reappraisal regulation technique is positively correlated with UF FA in healthy individuals^35,36^, consistent with results of meta-analyses implicating the OFC in this emotion regulation strategy^32^. In the present study, women with CD + AD and those with AD-only showed similar abnormalities of the UF. Yet the two disorders show distinct behavior patterns in response to threat, consistent with our finding of distinct correlates of the UF abnormality among women with AD-only and those with CD + AD. In the CD + AD women who show both approach and avoidance behaviors in response to threat, UF integrity was associated with the combination of poor anger control and anxiety symptoms, while among women with only ADs, who engage only in avoidance but not approach behaviors, UF integrity was associated with Harm Avoidance traits. These traits were measured in adolescence, a critical period for white matter development^97^, and have been shown to be stable into adulthood^98,99^. The finding of distinct correlates of the same UF abnormality in each disorder indirectly supports the hypothesis that this abnormality contributes to heightened sensitivity to threat and that additional characteristics such as harm avoidance and poor anger control play a role in learning to avoid or learning to behave aggressively. Aggressive behavior characterizes most toddlers, peaking at around age four and thereafter declining^100^. Children who observe aggressive behavior among parents, or who are not sanctioned for their own aggressive behavior, will likely continue to behave aggressively as they age^101^. This would be especially true if they displayed hyper-vigilance to threat, poor anger control, and impulsivity. Other toddlers with similar hyper-vigilance to threat and a distinct temperament, may discover that threating individuals or situations can be avoided rather than attacked. This may explain the diverging developmental trajectories, yet longitudinal research with careful behavioral assessments is required.

Reduced functional amygdala-OFC connectivity was observed only in participants, regardless of group, who presented a current anxiety disorder. This is finding is consistent with past reports of reduced amygdala-OFC functional connectivity among individuals presenting with current ADs^49,50^. Importantly, the functional connectivity measure was collected during rest and not during a task that is presumed to rely on functional connectivity between the amygdala and OFC. This likely explains the lack of association between UF integrity and functional connectivity measures, and lack of a linear association between the functional connectivity measure and current anxiety symptoms. Observing structural and not functional connectivity impairments in individuals with a history of ADs and no current ADs may be interpreted to suggest that reduced UF integrity is a consequence of previous episodes of ADs that render individuals vulnerable to future episodes and thereby contribute to the high rates of AD recurrence^102^, and to the lifetime stability of antisocial behavior^21^.

### Clinical implications

Our findings highlight the importance of diagnosing and treating comorbid ADs in adolescents presenting antisocial behavior and not viewing internalizing and externalizing behaviors as mutually exclusive. The abnormality of the UF was associated with disorders that occurred initially, in most cases, by adolescence. This abnormality may contribute to the stability of antisocial behavior and the recurrence of ADs. Longitudinal research is needed to determine whether treatment in adolescence that successfully reduces symptoms of ADs and conduct problems will also lead to healthy development of the UF, consistent with a recent study that observed changes to UF integrity among individuals with social anxiety disorder who responded positively to CBT^103^. Additionally, the results of the present study may be interpreted to suggest that individuals with CD require two distinct treatments: one aimed at decreasing hyper-reactivity to threat and another aimed at learning not to engage in aggressive behavior. Several studies provide evidence that children with comorbid anxiety and externalizing behaviors show similar improvement after anxiety treatment as children with only anxiety^104,105^. Additionally, one study reported that a treatment program addressing only anxiety was as effective in decreasing both anxiety and aggressive behavior in children as a treatment program that targeted both anxiety and aggressive behavior^106^. These positive treatment effects may have been achieved by reducing general hyper-reactivity to threat. However, the recommended treatment for CD is training parents to track and sanction their children’s inappropriate behaviors, yet whether such programs have long-term effects in preventing adult antisocial behavior remains largely unknown^107^. Future research on CD is required to determine whether a dual-treatment approach – reducing threat reactivity and thereby increasing UF structural integrity and learning not to behave aggressively – would more effectively reduce antisocial behavior and its persistence into adulthood.

Strengths of the current study include a well-characterized sample, and multi-modal imaging, including the use of the gold-standard technique for extracting proxy measures of UF structural integrity. Since we included only females, there was no confounding effect of sex. Additionally, studying young adult women with prospectively assessed CD during adolescence rules out confounding effects of pubertal development, known to occur earlier in girls with than without CD^5^, and to impact white matter development^97^. An obvious limitation of the current study is the lack of a CD-only group. This limited our ability to determine whether CD-only is associated with decreased UF integrity. Importantly, although there was a CD-only group at baseline when participants were adolescents^77^, almost all went on to develop an AD within seven years. This result is consistent with prospective, longitudinal studies showing that CD is an antecedent of ADs in adulthood^108,109^, and supports the notion that these two disorders share some neurobehavioral mechanisms. A second limitation is the use of the tensor model, which is vulnerable to intra-voxel crossing fibers, to reconstruct the UF for tractography. Although more advanced reconstruction methods such as spherical deconvolution would have been preferable, the tensor model was successful in reconstructing the UF with anatomical accuracy. Third, the exploratory analyses were undertaken with small samples and with measures designed for other purposes, underlining the necessity of replication of these results. Fourth, we cannot rule out the possibility that the three-day wash-out period for alcohol and drugs, confirmed using breathalyzer and saliva indicators prior to scan, may not have been sufficient to fully avoid the confounding effects of recent drug use. This limitation, however, applies primarily to the functional imaging findings; structural imaging is less susceptible to confounding effects of recent alcohol and drug use, while more susceptible to confounding effects of long-term use, such as that indexed by a dependence diagnosis. Our structural findings survived correction for lifetime alcohol and drug dependence. Excluding the four participants taking psychoactive medication at time of scan did not alter the functional connectivity results. A final limitation is that no neuropsychological and no task-fMRI data relevant to emotional regulation, reward delay discounting, reversal learning, or threat detection, interpretation and response were available.

## Conclusions

Young adult women with a history of conduct disorder and anxiety disorders showed the same reduced structural integrity of the UF as women with a history of only anxiety disorders, compared to women with no history of either disorder. This abnormality may be a marker of ADs or alternatively, it may explain, at least in part, the increased sensitivity to threat characterizing both antisocial and anxiety disorders. Exploratory analyses identified distinct correlates of the UF abnormality for each disorder: UF integrity was associated with high levels of the temperament trait of Harm Avoidance among women with AD-only, and with a combination of poor anger control and anxiety symptoms among women with CD + AD. A serendipitous finding was that in a clinical sample of teenage girls presenting with CD, almost all either presented a comorbid AD, or developed an AD in the subsequent seven years.

## Electronic supplementary material

Supplementary material
