# Supplementary material for: Amygdala-orbitofrontal structural and functional connectivity in females with anxiety disorders, with and without a history of conduct disorder
Source: Sci Rep. 2018 Jan 18;8:1101. doi: 10.1038/s41598-018-19569-7 (PMC5773614; doi:10.1038/s41598-018-19569-7)
Supplement: Supplementary file 1 — Supplementary material [file 41598_2018_19569_MOESM1_ESM.pdf]

Supplementary material for:

Amygdala-orbitofrontal structural and functional connectivity in females with anxiety disorders, with and without a history of conduct disorder

Philip Lindner[1,2,3]*

Pär Flodin[4,5]

Peter Larm[6]

Meenal Budhiraja[1]

Ivanka Savic-Berglund[7,8]

Jussi Jokinen[1,9]

Jari Tiihonen[1,10]

Sheilagh Hodgins[1,11]

[1]Department of Clinical Neuroscience, Karolinska Institutet, Stockholm, Sweden

[2]Stockholm Center for Dependence Disorders, Stockholm County Council, Stockholm, Sweden

[3]Department of Psychology, Stockholm University, Stockholm, Sweden

[4]Umeå Center for Functional Brain Imaging, Umeå University, Umeå, Sweden

[5]Center for Aging and Demographic Research, Umeå University, Umeå, Sweden

[6]Centre for Clinical Research, Uppsala University, Sweden

[7]Department of Women's and Children's Health, Karolinska Institutet, Stockholm, Sweden

[8]Neurology Clinic, Karolinska University Hospital, Huddinge, Sweden

[9]Department of Clinical Sciences, Umeå University, Umeå, Sweden

[10]Department of Forensic Psychiatry, University of Eastern Finland, Niuvanniemi Hospital, Kuopio, Finland

[11]Département de Psychiatrie, Université de Montréal, Montréal, QC, Canada

***Corresponding author:**

Philip Lindner

Frescati Hagväg 8, 114 19, Stockholm, Sweden

philip.lindner@psychology.su.se

+46 70 452 25 87

Supplementary Table S1. Frequency of anxiety disorders

Disorder	AD-only group (n=30)		AD+CD group (n=23)		Fisher exact statistic	
	Lifetime	Current	Lifetime	Current	Lifetime	Current
Agoraphobia	3%	0%	9%	4%	p=.57	p=.43
Generalized anxiety disorder	7%	7%	9%	9%	p=1	p=1
Obsessive compulsive disorder	10%	0%	26%	9%	p=.15	p=.18
Panic disorder	33%	3%	43%	13%	p=.57	p=.31
Post-traumatic stress disorder	17%	3%	26%	0%	p=.5	p=1
Social anxiety disorder	50%	3%	35%	9%	p=.4	p=.57
Specific phobia	33%	7%	61%	22%	p=.06	p=.22
Substance-induced anxiety disorder	7%	0%	4%	0%	p=1	NA
Anxiety disorder not otherwise specified	20%	0%	26%	4%	p=.74	p=.43
Anxiety disorder due to somatic illness	0%	0%	0%	0%	NA	NA
More than one anxiety disorder	43%	0%	65%	9%	p=.17	p=.18

More than two anxiety disorders	27%	0%	35%	9%	p=.56	p=.18
More than three anxiety disorders	7%	0%	26%	9%	p=.06	p=.18
